# CSF chitinases before and after symptom onset in amyotrophic lateral sclerosis

**DOI:** 10.1002/acn3.51114

**Published:** 2020-07-14

**Authors:** Elizabeth Gray, Alexander G. Thompson, Joanne Wuu, Joe Pelt, Kevin Talbot, Michael Benatar, Martin R. Turner

**Affiliations:** ^1^ Nuffield Department of Clinical Neurosciences University of Oxford Oxford UK; ^2^ Department of Neurology University of Miami Miami Florida

## Abstract

**Objective:**

To evaluate the CSF levels of chitinase proteins during the presymptomatic and early symptomatic phases of amyotrophic lateral sclerosis (ALS).

**Methods:**

CSF samples were obtained from 16 controls, 55 individuals at‐risk for ALS (including 18 carrying a mutation in *C9ORF72*, 33 in *SOD1*), 12 ALS patients, and 7 phenoconverters (individuals diagnosed with ALS during follow‐up). At‐risk individuals and phenoconverters were enrolled through the *Pre‐fALS* study, which includes individuals carrying an ALS‐associated gene mutation without disease manifestations at initial assessment. Longitudinal CSF collections, where possible, took place every 3‐12 months for ALS patients and every 1‐2 years for others. CSF levels of chitotriosidase 1 (CHIT1), chitinase‐3‐like protein 1 (CHI3L1, YKL‐40) and chitinase‐3‐like protein 2 (CHI3L2, YKL‐39) were measured by ELISA, along with CHIT1 activity. Longitudinal changes in at‐risk individuals and phenoconverters were fitted to linear mixed effects models.

**Results:**

Slowly rising levels of CHIT1 were observed over time in the at‐risk individuals (slope 0.059 log_10_[CHIT1] per year, *P* < 0.001). Among phenoconverters, CHIT1 levels and activity rose more sharply (0.403 log_10_[CHIT1] per year, *P* = 0.005; 0.260 log_10_[CHIT1 activity] per year, *P* = 0.007). Individual levels of both CHI3L1 and CHI3L2 remained relatively stable over time in all participant groups.

**Interpretation:**

The CHIT1 neuroinflammatory response is a feature of the late presymptomatic to early symptomatic phases of ALS. This study does not suggest a long prodrome of upregulated glial activity in ALS pathogenesis, but strengthens the place of CHIT1 as part of a panel of biomarkers to objectively assess the impact of immune‐modulatory therapeutic interventions in ALS.

## Introduction

Inflammatory cell infiltration is a consistent neuropathological feature of the neurodegenerative disorder amyotrophic lateral sclerosis (ALS).^1^, ^2^, ^3^, ^4^ *In vivo* microglial activity has also been demonstrated using positron emission tomography (PET)^5^ and through analysis of cerebrospinal fluid (CSF) cytokines and proteins secreted by microglia.^6^ The precise role of glia in the pathophysiology of ALS is a matter of controversy. It remains unresolved whether microglia are involved in the initiation of motor neuron degeneration, are an exacerbating factor promoting disease progression, or even exert a neuroprotective effect.^7^

A set of glial biomarkers, the chitinase proteins, have been shown to be elevated in the CSF of ALS patients using proteomic analysis and subsequently validated using immunoassays. The different chitinase proteins reflect different facets of microglial and astroglial activity in ALS.^8^, ^9^, ^10^, ^11^, ^12^, ^13^, ^14^, ^15^ At least 10% of all cases of ALS are attributable to mutations in one of four genes: *C9ORF72*, *SOD1*, *TARDBP,* and *FUS*. Studying the carriers of these genetic mutations provides a unique opportunity to define the biochemical landscape preceding the development of clinically manifest neurodegeneration.^16^ Analysis of samples from such individuals has demonstrated rising CSF levels of the axonal degeneration markers neurofilament light chain (NfL)^17^ and phosphorylated neurofilament heavy (pNfH)^18^ in the months prior to the emergence of symptomatic disease.

In this study, we sought to measure CSF chitinase levels in a similar population of carriers of ALS‐causing genetic mutations in the presymptomatic period, during the transition to symptomatic disease (i.e. phenoconversion), and in the symptomatic phase of the disease, to explore the temporal course of glial responses in relationship to the emergence of clinically manifest ALS.

## Materials and Methods

### Participant characterization and sampling

The “Pre‐Symptomatic Familial ALS” study (*Pre‐fALS*, registered on clinicaltrials.gov: NCT00317616) is a longitudinal natural history and biomarker study of asymptomatic carriers of ALS‐causing gene mutations. *Pre‐fALS* participants were recruited from across North America and underwent an in‐depth series of assessments in person every 1–2 years, which included CSF sampling (if participant willing) using established standard operating procedures. Control participants were similarly evaluated, and CSF collected every 1–2 years, and ALS patients every 3–12 months.

In addition, *Pre‐fALS* participants were followed quarterly by telephone. Those who developed symptoms or signs suggestive of disease were followed with more frequent in‐person assessments as needed, depending on the evolution of symptoms over time, and were classified as phenoconverters once definite symptoms or signs of disease emerged.^19^ Longitudinal data are therefore acquired, to the extent possible, in the presymptomatic phase, around the time that symptoms begin to emerge (peri‐symptomatic), and following phenoconversion, defined as the emergence of definite symptoms or signs (clinical or electromyographic) that clearly indicate manifest disease.^19^ Since participants were sometimes reluctant to undergo lumbar puncture, however, both pre and post‐conversion CSF was not always available. Among the 10 phenoconverters, presymptomatic CSF was only available from 7 (shown as “converters” in Table 1; the three phenoconverters in whom CSF was only available after phenoconversion are included in the “ALS” group in Table 1 and 2). Moreover, both pre‐ and post‐conversion CSF was available from five converters (Table 2). Among the samples included in this experiment, there were no phenoconverters from whom multiple preconversion samples as well as at least one postconversion CSF sample were available.

**Table 1:** Participant characteristics (all participants).

	Control	At‐risk	Converter	ALS
*n*	16	55	7	12
Baseline age, years; mean ± SD^1^	46.0 ± 11.3	47.4 ± 11.4	55.0 ± 14.5	57.5 ± 7.2
Male, *n* (%)^2^	7 (43.8)	17 (30.9)	5 (71.4)	6 (50.0)
Gene, *n*				
*SOD1* A4V	–	18	4	2
*SOD1* non‐A4V	–	15	0	1
*C9ORF72*	–	18	2	2
Other	–	4	1	0
Unknown	–	0	0	7
Onset site, *n*				
Bulbar	–	–	0	2
Limb	–	–	5	9
Other	–	–	1	1
Unknown	–	–	1	0
Baseline time from onset, months; median [IQR]	–	–	−12.1 [−22.9 to −9.3]	38.30 [24.8 to 60.3]
Baseline time from diagnosis, months; median [IQR]	–	–	−13.3 [−25.0 to −9.9]	13.0 [7.8 to 22.2]
Baseline ALSFRS‐R, median [IQR]	–	–	–	39.0 [35.5 to 44.5]
Baseline ∆FRS, median [IQR]	–	–	–	0.31 [0.21 to 0.57]

ALS, amyotrophic lateral sclerosis; SD, standard deviation; IQR, interquartile range; ALSFRS‐R, revised ALS functional rating scale; ∆FRS, estimated ALSFRS‐R progression based on baseline ALSFRS‐R, points per month.

^1^ Kruskal‐Wallis *H*‐test *P* = 0.024 (Dunn’s post hoc ALS vs control *P* = 0.0178, ALS vs. at‐risk *P* = 0.025).

^2^ Fisher exact test *P* = 0.150.

**Table 2:** Participant characteristics (longitudinal subset).

	Control	At‐risk	Converter	ALS
*n*	4	36	5	7
Baseline age, years; mean ± SD^1^	45.9 ± 13.1	50.9 ± 9.5	55.7 ± 9.1	57.7 ± 8.06
Male, *n* (%)^2^	1 (25.0)	12 (33.3)	4 (57.1)	2 (40.0)
Number of visits, median; range	2 [2‐3]	2 [2‐7]	3 [3‐7]	2 [2‐6]
Duration of follow‐up, months; median [IQR]	26.5 [23.2‐30.7]	34.5 [23.3‐49.5]	22.1 [9.0‐32.4]	10.5 [9.6‐26.5]
Gene, *n*				
*SOD1* A4V	–	13	2	1
*SOD1* non‐A4V	–	10	0	1
*C9ORF72*	–	10	2	2
Other	–	3	1	0
Unknown	–	0	0	3
Onset site, *n*				
Bulbar	–	–	0	1
Limb	–	–	3	5
Other	–	–	1	1
Unknown	–	–	1	0
Baseline time from onset, months; median [IQR]	–	–	−22.3 [−23.4 to −8.5]	18.2 [6.3 to 31.6]
Baseline time from diagnosis, months; median [IQR]	–	–	−23.5 [−26.5 to −9.2]	13.0 [2.4 to 22.2]
Baseline ALSFRS‐R, median [IQR]	–	–	–	41.5 [37.5 to 44.8]
Baseline ∆FRS, median [IQR]	–	–	–	0.56 [0.34 to 1.01]

ALS, amyotrophic lateral sclerosis; SD, standard deviation; IQR, interquartile range; ALSFRS‐R, revised ALS functional rating scale; ∆FRS, estimated ALSFRS‐R progression based on baseline ALSFRS‐R, points per month.

^1^ Kruskal‐Wallis *H*‐test *P* = 0.170;

^2^ Fisher exact test *P* = 0.231.

The study was approved by the University of Miami Institutional Review Board, and all participants provided written informed consent.

### Sample Collection, Processing, and Storage

CSF (free of macroscopic hemoglobin) was collected in polypropylene tubes, centrifuged (1750 *g* for 10 min at 4°C), aliquoted using a sterile pipette into precapped polypropylene cryogenic sterile freestanding conical microtubes, frozen within 15–20 min of collection, and stored at −80°C. Samples were subjected to a single freeze‐thaw cycle prior to use.

### Biochemical assays

All ELISAs and activity assays were performed in duplicate. Plates were read using a FLUOstar Omega plate reader (BMG LABTECH, UK). Standard curves were fitted with 4‐parameter logistic regression using MARS data analysis software (BMG LABTECH, UK). CSF samples were thawed on ice prior to measurement. Measurements of CSF concentrations and activity were performed using commercially available assays according to the manufacturer’s instructions (CircuLex human Chitotriosidase ELISA, CircuLex human YKL‐39 ELISA, CycLex Chitotriosidase Fluorimetric Assay Kit, MBL, UK; Human Chitinase‐3‐like 1 Quantikine ELISA kit, R&D systems, UK).

Samples were diluted where necessary to achieve a concentration within the linear range of standard curve measurements. Median intraassay and interassay coefficients of variation were below 10% for all assays: CHIT1 intraassay median 6.9% (IQR 2.9%–15.6%), interassay 4.0% (2.4%–5.2%), lower limit of detection (LLOD) 48.3 pg/mL; CHI3L1 5.8% (2.4%–12.09%), 2.6% (1.4%–11.9%), LLOD 3.55 pg/mL; CHI3L2 3.5% (1.2–7.3), 3.0% (0.7%–6.2%), LLOD 35.1 pg/mL; and CHIT1 activity 6.3% (1.7%–13.9%), 1.58% (0.8%–2.5%), LLOD 12.0 nmol/h/mL. Samples for which the analyte value was unmeasurably low were imputed with the LLOD.

### Statistical analysis

Statistical analysis was performed in R. Baseline age and sex were compared between groups using the Kruskal‐Wallis *H*‐test (with Dunn test for *post hoc* comparisons) and Fisher’s exact test, respectively. Chitinase protein concentrations were log (base 10)‐transformed prior to analysis. Comparisons of baseline analyte concentration between groups were performed using analysis of covariance (ANCOVA) controlling for age, with *post hoc* Tukey’s honestly significant difference. For CHI3L2, residuals were nonnormal with and without log transformation, so ANCOVA by ranks was performed.

Longitudinal analysis of analytes was performed using a random intercept, random slope linear mixed effects model with unstructured covariance structure and degrees of freedom as described by Pinheiro and Bates.^20^ Only participants with more than one visit were included, and the model adjusted for baseline age. Separate analyses were performed for the four groups. For the control and at‐risk groups, time was anchored to the date of the baseline visit, whereas for the phenoconverter and ALS groups, time was anchored to the date of phenoconversion (first reported symptoms or observed signs that definitely indicate disease). In addition, among phenoconverters, analyte levels immediately before and after phenoconversion were compared using a paired *t*‐test. All hypothesis tests were two‐sided (*P* < 0.05).

## Results

### Demographics

Cohort demographics are summarized in Table 1 (entire cohort) and Table 2 (longitudinal subset). Participants with ALS were significantly older than healthy control (*P* = 0.018) or at‐risk participants (*P* = 0.023). The groups did not differ significantly by sex (3‐group comparison, *P* = 0.15).

### Cross‐sectional analysis

#### Association of baseline chitinase levels and age

**Figure 1:**
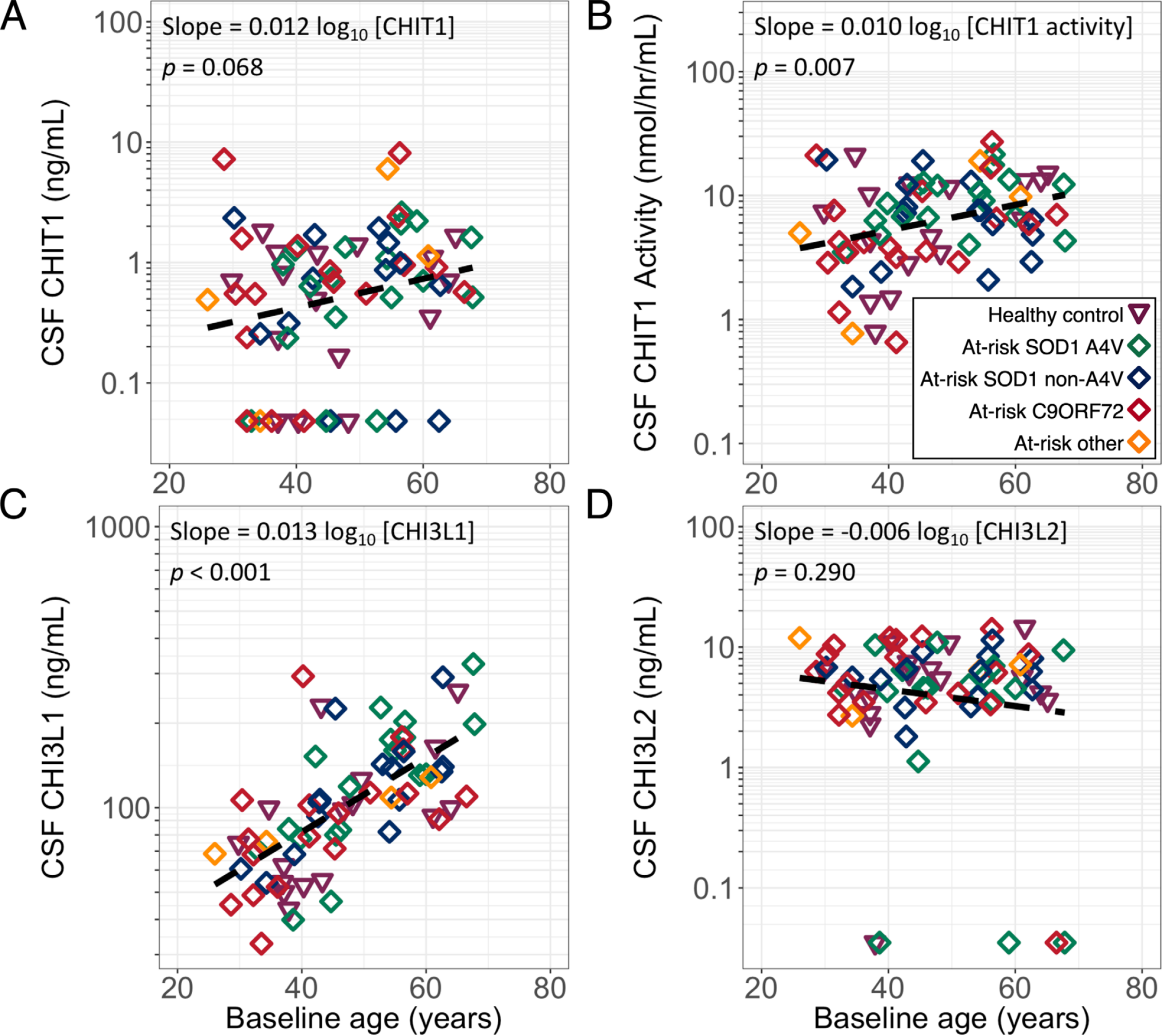
(A) CHIT1, (B) CHIT1 activity, (C) CHI3L1, (D) CHI3L2 by baseline age. Dashed line indicates linear regression including all data. CSF, cerebrospinal fluid; CHIT1, Chitotriosidase 1; CHI3L1, Chitinase 3‐like protein 1; CHI3L2, Chitinase 3‐like protein 2; HC, healthy control; At‐risk, presymptomatic gene carrier; C9ORF72, *C9ORF72* hexanucleotide repeat expansion carrier; A4V, *SOD1* A4V mutation carrier; non‐A4V, *SOD1* non‐A4V mutation carrier; none, no mutation; Other, carrier of other highly penetrant ALS‐causing gene mutation.

Linear models of baseline chitinase protein levels and baseline age were constructed. Due to the strong association between ALS and chitinase levels, the ALS and converter groups were excluded from this analysis to avoid confounding the relationship between age and chitinase levels. There was an association of baseline age with CHIT1 activity and CHI3L1 levels, but not for CHIT1 or CHI3L2 levels (Fig. 1).

#### Baseline analyte levels across groups

**Figure 2:**
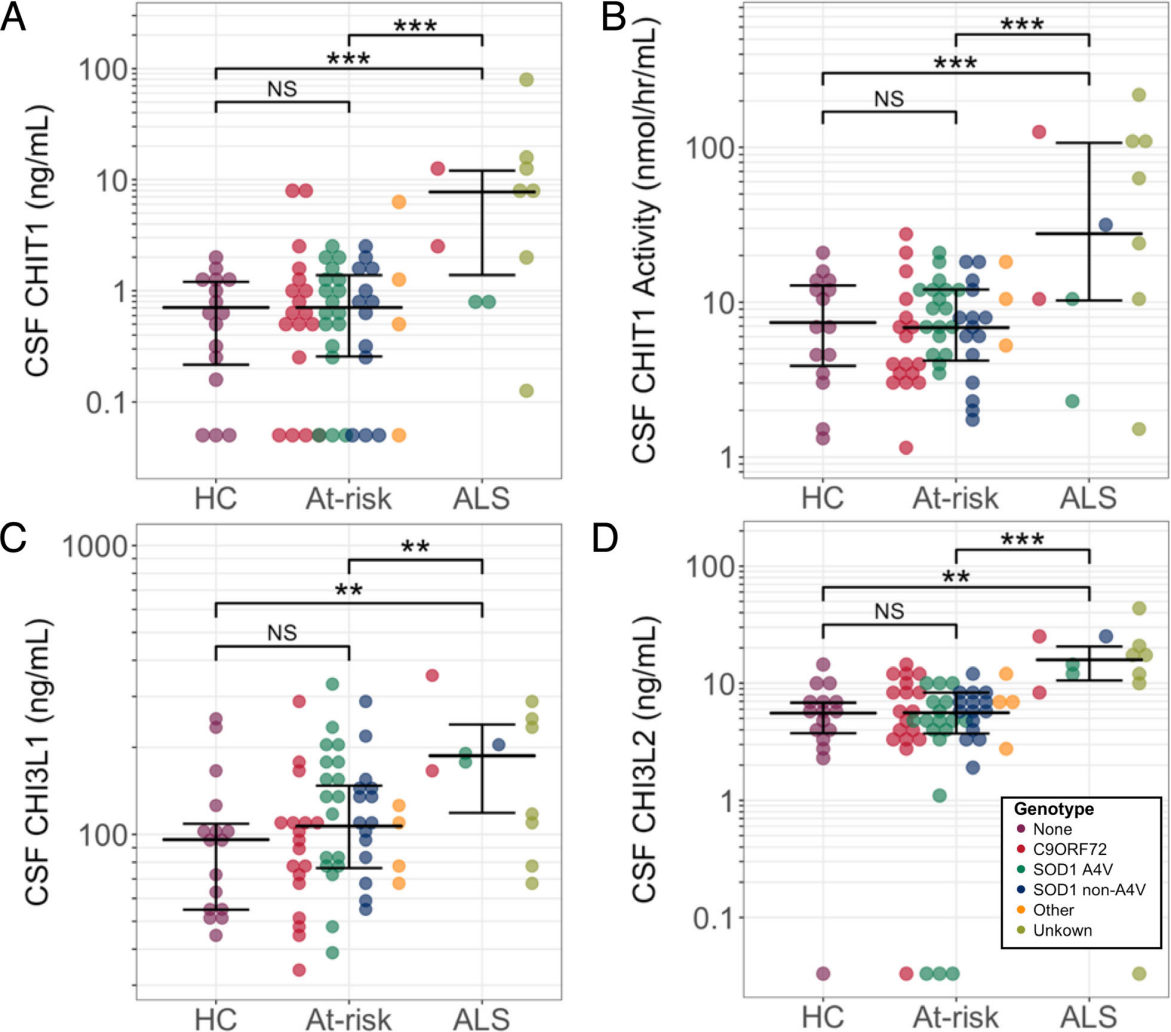
(A) CHIT1 concentration, (B) CHIT1 activity, (C) CHI3L1 concentration, (D) CHI3L2 concentration. Comparisons between groups were performed with all genotypes combined within each group. **Multiple comparison‐corrected *P* < 0.01, ***P < 0.001. CSF, cerebrospinal fluid; CHIT1, Chitotriosidase 1; CHI3L1, Chitinase 3‐like protein 1; CHI3L2, Chitinase 3‐like protein 2; HC, healthy control; At‐risk, presymptomatic gene carrier; ALS, amyotrophic lateral sclerosis; C9ORF72, *C9ORF72* hexanucleotide repeat expansion carrier; A4V, *SOD1* A4V mutation carrier; Non‐A4V, *SOD1* non‐A4V mutation carrier; none, no mutation; Other, carrier of other highly penetrant ALS‐causing gene mutation; Unknown, no mutation identified; NS, not statistically significant.

**Figure 3:**
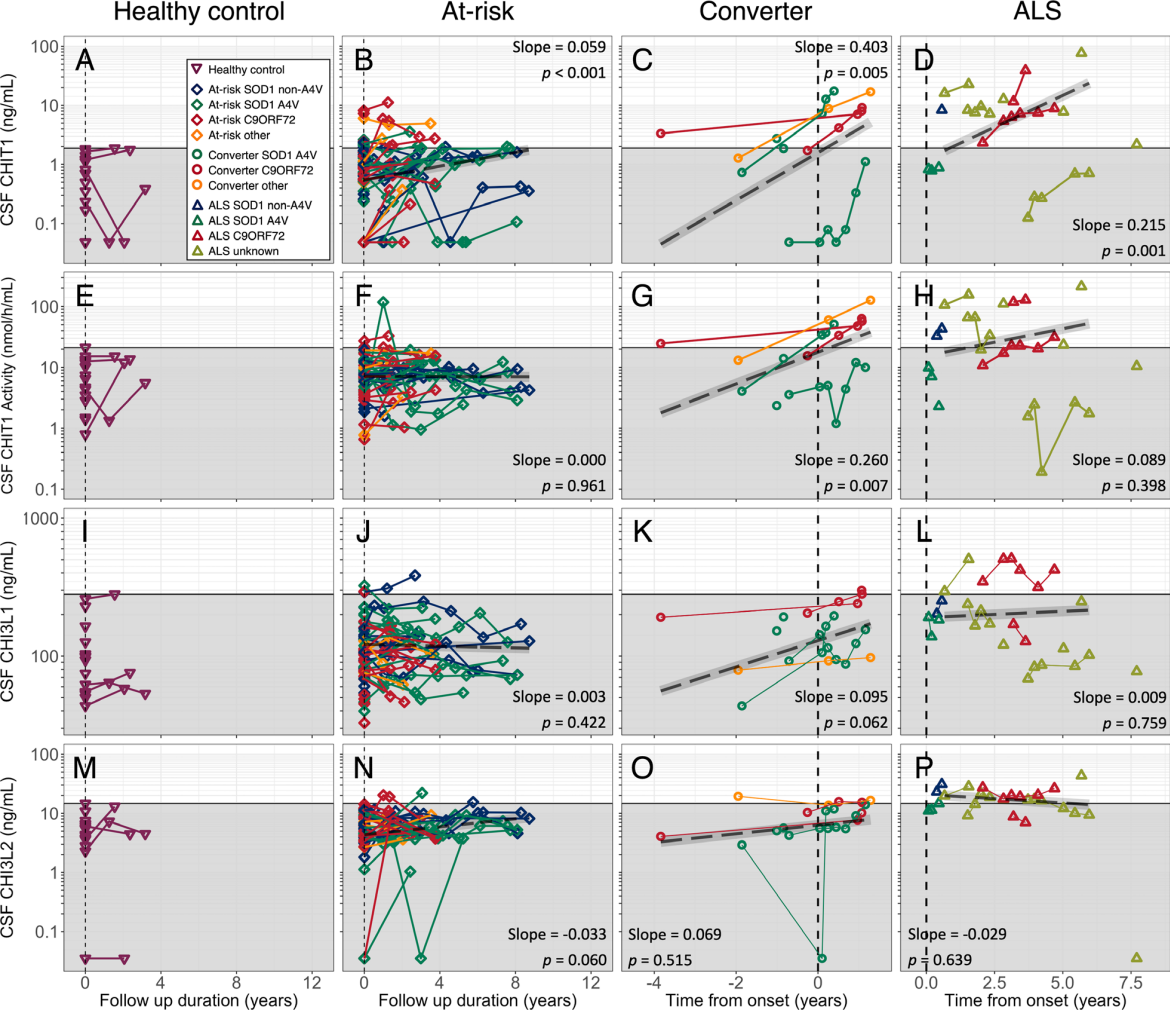
(A–D) CHIT1 concentration, (E–H) CHIT1 activity, (I–L) CHI3L1 concentration, (M–P) CHI3L2 concentrations; (A, E, I, M) healthy controls; (B, F, J, N) presymptomatic at‐risk individuals; (C, G, K, O) participants developing ALS during follow‐up and (D, H, L, P) participants with symptoms of ALS at enrolment. Grey box indicates highest level of each analyte (or activity) detected in control samples. Dashed black line with grey ribbon indicates the estimated trajectory of chitinase protein levels in asymptomatic carriers (B, E, H, L) or converters and ALS patients (C, F, I, L), with slope and *P*‐value obtained from the linear mixed effect model and protein level calculated using the median baseline age in that group. Vertical dashed line indicates first sampling (healthy control and at‐risk individuals), or date of onset of clinically manifest disease (phenoconverter and ALS groups). CSF, cerebrospinal fluid; CHIT1, Chitotriosidase 1; CHI3L1, Chitinase 3‐like protein 1; CHI3L2, Chitinase 3‐like protein 2; At‐risk, presymptomatic gene carrier; ALS, amyotrophic lateral sclerosis; Converter, participants asymptomatic at enrolment developing clinically manifest disease during follow‐up; C9ORF72, *C9ORF72* hexanucleotide repeat expansion carrier; A4V, *SOD1* A4V mutation carrier; Non‐A4V, *SOD* *non‐A*4V mutation carrier; None, no mutation; Other, carrier of other highly penetrant ALS‐causing gene mutation; Unknown, no mutation identified.

Baseline levels of CHIT1, CHI3L1, CHI3L2, and CHIT1 activity were significantly higher in participants with symptomatic ALS than controls and asymptomatic gene carriers, controlling for age. Although in some at‐risk participants baseline levels of CHIT1, CHIT1 activity and CHI3L1 exceeded the upper end of the range recorded in control samples (Fig. 3, shaded area), there was no significant difference in the levels of any analyte between controls and asymptomatic gene carriers (Fig. 2A–D; Table 3). There were no significant differences within groups for different genotypes (Table 4).

**Table 3:** Baseline chitinase concentration and activity by group.

	Median value			Pair‐wise comparisons^1^		
	HC	At‐risk	ALS	At‐risk vs. HC	ALS vs. HC	ALS vs. at‐risk
CHIT1 (ng/mL)	0.71	0.71	7.75	0.890	<0.001	<0.001
*N* (*n* LLOD)	16 (3)	53 (11)	11 (0)			
CHIT1Act (nmol/hr/mL)	6.93	6.76	28.08	0.923	<0.001	<0.001
*N* (*n* LLOD)	16 (0)	55 (1)	12 (0)			
CHI3L1 (ng/mL)	95.81	106.69	187.06	0.364	0.001	0.002
*N* (*n* LLOD)	16 (0)	55 (0)	12 (0)			
CHI3L2 (ng/mL)	5.56	5.60	15.87	0.981	0.001	<0.001
*N* (*n* LLOD)	16 (1)	55 (4)	12 (1)			

ALS, amyotrophic lateral sclerosis; HC, healthy control; CHIT1, Chitotriosidase 1; CHI3L1, Chitinase 3‐like protein 1; CHI3L2, Chitinase 3‐like protein 2; LLOD, lower limit of detection; *N*, total number (including number of LLOD values); *n* LLOD, number of LLOD values.

^1^ ANCOVA (by ranks for CHI3L2) controlling for age, with *post hoc* Tukey HSD.

**Table 4:** Cross‐sectional analysis of CSF chitinase levels and activity within at‐risk group comparing genotypes.

	*N*	Median (ng/mL)	*P*‐values^1^		
			*C9ORF72*	*SOD1* A4V	*SOD1* non‐A4V
CHIT1 level					
*C9ORF72*	18	0.63	–	–	–
*SOD1* A4V	18	0.72	>0.999	–	–
*SOD1* non‐A4V	13	0.74	0.988	0.983	–
Other	4	0.81	0.998	0.999	0.981
CHIT1 activity					
*C9ORF72*	18	4.21	–	–	–
*SOD1* A4V	18	8.86	0.188	–	–
*SOD1* non‐A4V	15	7.23	0.829	0.696	–
Other	4	7.38	>0.999	0.665	0.971
CHI3L1					
*C9ORF72*	18	93.23	–	–	–
*SOD1* A4V	18	130.93	0.119	–	–
*SOD1* non‐A4V	15	106.73	0.26	0.988	–
Other	4	92.17	0.998	0.621	0.754
CHI3L2					
*C9ORF72*	18	6.2	–	–	–
*SOD1* A4V	18	4.58	0.347	–	–
*SOD1* non‐A4V	15	6.25	0.994	0.261	–
Other	4	6.76	0.988	0.548	0.999

ALS, amyotrophic lateral sclerosis; HC, healthy control; CHIT1, Chitotriosidase 1; CHI3L1, Chitinase 3‐like protein 1; CHI3L2, Chitinase 3‐like protein 2; LLOD, lower limit of detection; *N*, total number.

^1^ ANCOVA (by ranks for CHI3L2) controlling for age, with *post hoc* Tukey HSD.

### Longitudinal analysis

The longitudinal trajectories of CHIT1, CHIT1 activity, CHI3L1 and CHI3L2 over time are illustrated in Figure 3.

In the at‐risk group, we observed a gradual increase in CSF CHIT1 levels of 0.059 log_10_ [CHIT1] per year (*P* < 0.001), but no significant change in CHIT1 activity, CHI3L1 or CHI3L2 levels. The small number of longitudinal control samples available precluded a similar analysis in this group.

Modeling analyte levels across the transition from presymptomatic to symptomatic ALS (*n* = 5, 3–7 observations per participant for each analyte), there was an increase in CSF CHIT1 levels of 0.403 log_10_ [CHIT1] per year (*P* = 0.005; Fig. 3C), and CHIT1 activity 0.260 log_10_ [CHIT1 activity] per year (*P* = 0.007; Fig. 3G). No significant changes were observed in CHI3L1 (0.095 log_10_[CHI3L1] per year, *P* = 0.062, Fig. 3K) or CHI3L2 (slope 0.069 log_10_ [CHI3L2] per year, *P* = 0.515; Fig. 3O). Comparing levels from the last sample taken before phenoconversion (median [range] = 22.3 [46.1–3.1] months prior to symptom onset) and the first sample taken after phenoconversion (3.1 [0.5–11.6] months after symptoms onset) showed an increase in CHIT1 (*P = *0.042, paired *t*‐test), CHIT1 activity (*P* = 0.031) and CHI3L1 (*P* = 0.006), but not in CHI3L2 levels (*P* = 0.88).

In participants with symptomatic ALS (*n* = 7, 2–6 observations per participant for each analyte), there was an increase in CSF CHIT1 over time (0.215 log_10_ [CHIT1] per year, *P* = 0.001; Fig. 3D). No significant longitudinal changes in CHIT1 activity, CHI3L1 or CHI3L2 level were observed in this group (0.089 log_10_ [CHIT1 activity] per year, *P* = 0.398 Fig. 3H; 0.009 log_10_ [CHI3L1] per year, *P* = 0.759, Fig. 3L; −0.029 log_10_ [CHI3L2] per year, *P* = 0.639, Fig. 3P).

## Discussion

Previous cross‐sectional studies have shown elevated CSF chitinase protein levels in ALS patients versus controls and no elevation in asymptomatic gene carriers compared with healthy controls.^11^, ^12^ The cross‐sectional analysis in this study concurs with these findings, showing levels of CHIT1, CHI3L1 and CHI3L2 concentrations, as well as CHIT1 activity, to be similar in healthy controls and at‐risk individuals who have not yet phenoconverted (irrespective of genotype), but elevated in symptomatic ALS compared to both healthy controls and at‐risk individuals. Although baseline levels of CHIT1, CHIT1 activity and CHI3L1 in some at‐risk participants were higher than the maximum healthy control level, this did not foretell imminent phenoconversion.

The longitudinal data presented begin to define the temporal profile of the chitinase response in ALS. There is a gradual rise in CSF CHIT1 protein in at‐risk participants during the presymptomatic period followed by a rapid rise in CHIT1 levels (0.260 log_10_[CHIT1] per year) and activity between the late presymptomatic and early symptomatic phases of disease among phenoconverters followed by a more gradual increase in CHIT1 levels during the symptomatic period. These data suggest that microglia are relatively quiescent in the years preceding transition to symptomatic ALS, then increase in activity rapidly in the peri‐symptomatic period before abating during the symptomatic period. Notably the rise in CHIT1 level in at‐risk participants over time was greater than would be expected for age based on our cross‐sectional analysis of the association between CHIT1 and age (0.059 vs 0.010 log_10_[CHIT1] per year). This is consistent with low‐level microglial activity occurring in ALS gene carriers in the years before symptom onset, though additional longitudinal analysis of healthy control subjects is required to confirm that the trajectory of rising CHIT1 differs between at‐risk participants and healthy controls. We also acknowledge the limitations of the small sample size in relation to estimating the magnitude of changes in chitinase protein levels in the converter and symptomatic ALS groups.

This study did not demonstrate the sort of changes in CHI3L1 and CHI3L2 that we observed in CHIT1 protein and activity across the transition from presymptomatic to symptomatic ALS. This may be a consequence of the greater variability in the levels of these proteins and the small number of participants for whom longitudinal samples are available spanning this transition (five participants, 20 samples in total). Alternatively, this might reflect differing longitudinal profiles of these proteins and the underlying processes they represent. Studies on frontotemporal dementia, ALS and Alzheimer’s disease suggest that CHIT1 reflects microglial activity and is associated with the rate of disease progression in ALS,^9^, ^11^, ^12^ while histopathological work in Alzheimer’s disease, and recently ALS, suggests that CHI3L1 is expressed preferentially by astrocytes^14^, ^21^ and studies in ALS suggest that CHI3L1 is more closely associated with clinical features of central dysfunction denoted by upper motor neuron involvement and degree of cognitive dysfunction.^11^, ^12^, ^14^ Further work is required to confirm these findings and refine our understanding of the pathological changes underlying variation in these three proteins.

*CHIT1* polymorphisms have been associated with lower expression and activity of CHIT1 in other disease settings,^22^ and this was not explored in this study population. A previous study suggested that such polymorphisms are not associated with meaningful differences in chitinase levels in ALS patient studies.^11^

Since we do not (yet) have multiple longitudinal CSF chitinase measurements within individual phenoconverters during the presymptomatic phase, we cannot be certain whether the increase we have observed in CHIT1 protein and activity across the transition to manifest disease reflects a late presymptomatic or early postsymptomatic phenomenon. This leaves unanswered the question of how the timing of the peri‐symptomatic increase in CHIT1 protein and activity relates to previously reported changes in neurofilaments. Serum NfL (and to a lesser extent pNfH) clearly increases prior to phenoconversion, as well as in the early symptomatic phase of the disease (observations that were made possible by the ease of neurofilament measurement in blood and the ready availability of longitudinal blood samples, including multiple time points pre and post‐phenoconversion).^17^, ^23^ The granularity of CSF sampling from longitudinal^17^ and cross‐sectional^24^ studies around the time of phenoconversion is insufficient to categorically conclude that the temporal profile of CSF neurofilament levels mirrors that of serum neurofilament levels.

Given the limitations of the current dataset, as noted above, we remain uncertain whether changes in CHIT1 are likely to reflect a “physiological” response to the neuronal loss evidenced by rising neurofilament levels, or whether increased microglial activity might be a trigger that precedes neurodegeneration.

The longitudinal profile of the chitinase proteins outlined in this study supports a model in which an accelerated increase in microglial activation, at least based on the surrogate marker of CHIT1 levels, occurs during the late presymptomatic or early symptomatic phases of disease, but is not a major feature of the years of asymptomatic life prior to the emergence of clinical manifestations of ALS.

Animal model work has primarily examined microglial activity in the *SOD1* ^G93A^ mouse and suggests that microglial activation is an early feature of ALS in this model, occurring before the onset of detectable neurodegeneration and increasing in later disease stages.^25^, ^26^ This is accompanied by changes in microglial morphology^27^ and cytokine profile through the disease course, with more antiinflammatory protective profiles in presymptomatic and early stages^28^ progressing to a more proinflammatory phenotype at later stages.^29^ Expression of human mutant SOD1 in mouse microglia specifically also appears to drive disease progression.^30^

ALS‐causing mutations in *TBK1*,^31^ encoding Tank binding kinase‐1, a protein involved in innate immune system modulation, and the development of autoimmunity in *C9ORF72*‐null mice^32^ (*C9ORF72* haploinsufficiency being one proposed pathogenic mechanism) support a role for the immune system in ALS pathogenesis, but mechanistic insights into microglial mechanisms are limited. The data presented here do not show major differences in the longitudinal profile of chitinases in relation to genotype, but the small number of subjects for each genotype limit interpretation.

Further study of the biochemical milieu during the presymptomatic phase and around the time of phenoconversion and the ongoing accrual of CSF samples from presymptomatic gene mutation carriers will enable more accurate profiling of inflammatory changes detectable in CSF to better define the temporal relationship between neurodegeneration and neuroinflammation. Development of reliable blood‐based assays, assuming that blood chitinase levels meaningfully reflect microglial responses in the CNS, would be advantageous. Meanwhile, this study further supports the use of chitinase proteins as potentially valuable markers of glial activity for candidate drugs targeting the neuroinflammatory response in the therapy of ALS.

## Conflicts of Interest

MB has a patent Appl. No. 16/616,853 pending and reports a grant from Eli Lilly.

## Author Contributions

All authors contributed to the study design. MB, EG, JP, JW, AGT, and MRT participated in the data acquisition and analysis. AGT and EG drafted the manuscript and figures. All authors critically reviewed and edited the manuscript.
